# Response to the commentary on “are all measures of liver ${\text{Kp}}_{\text{uu}}$ a function of $F_{H}$, as determined following oral dosing, or have we made a critical error in defining hepatic drug clearance?”

**DOI:** 10.1016/j.ejps.2025.107088

**Published:** 2025-03-31

**Authors:** Leslie Z. Benet, Jasleen K. Sodhi

**Affiliations:** Department of Bioengineering and Therapeutic Sciences, Schools of Pharmacy and Medicine, University of California, UCSF, 513 Parnassus Ave., Room S-822, San Francisco, CA 94143-0912, USA

**Keywords:** Kp_uu_, Hepatic bioavailability, Liver to blood ratio, Hepatic clearance, Extended clearance model, Protein binding

## Abstract

Recently, Sugano commented on our above-titled publication stating that he was a reviewer for the paper. In the Acknowledgements, Sugano indicated that we “kindly suggested publishing the reviewer report as an article”. What we had actually written was: “prior to accepting the [unidentified] reviewer’s approach, however, we believe he/she would have to publish a paper justifying the methodology and proposing how *in vivo* clinical data could be analyzed with such methodology”. We further explained the fallacy of the accepted mechanistic models of hepatic elimination in a subsequent manuscript titled “Pharmacokinetic theory must consider published experimental data”, published online a month after the Sugano commentary became available –not in time for the author to review the full explanation before his commentary was published. Our above-titled publication makes no assumptions or discussions regarding how $Kp_{uu}$ should be measured, the major topic of the Sugano paper. Here we detail why the commentary methodology is not relevant to our demonstration that the present mechanistic models of hepatic elimination all lead to the unlikely conclusion that $Kp_{uu}$ cannot exceed unity and is related to $F_{H}$. We also detail why the Extended Clearance Model should not be used for *in vitro*-*in vivo* extrapolation.

## 1. Introduction

Quoting from our most recent mini-review “Evaluating *in vivo* data for drug metabolism and transport: Lessons from Kirchhoff’s Laws” (Benet and Sodhi, 2024c). “In the last two years we recognized that the derivation of clearance for *in vivo* drug metabolism and transport processes in series and in parallel could be simply accomplished independent of differential equations utilizing Kirchhoff’s Laws from physics (Patcher et al., 2022; Benet and Sodhi, 2023; 2024b; 2024c; Wakuda et al., 2024). In fact, we demonstrated that traditional pharmacokinetic equations based on differential equations for drug absorption and elimination, utilized and taught for the past century, cannot accurately define the measured *AUC* when clearance of drug from the administration site is comparable to or less than clearance of an iv bolus dose. Thus, using Kirchhoff’s Laws we can explain why it is possible to obtain bioavailability from systemic concentration measurements that exceed unity, why renal clearance can be a function of drug input processes, and why statistically different bioavailability measures may be found for urinary excretion *versus* systemic concentration measurements in the same study (Wakuda et al., 2024). We discovered, in 2022, that [adaption of] Kirchhoff’s Laws from physics would provide a pathway to derive clearance and overall rate constants for in series processes, independent of differential equation derivations (Patcher et al., 2022). We showed that consistent with Kirchhoff’s Laws for processes in series, the inverse of the overall clearance would equal the sum of the inverse of the individual rate-defining processes entering and the inverse of the individual rate-defining process leaving.”

A rate-defining process is one that (a) must have a positive value, (b) that on its own, could potentially define total clearance, and (c) one that is possible to measure experimentally *in vivo* when it solely determines the clearance. When hepatic basolateral transporters are not relevant for hepatic clearance, the entering rate-defining process is hepatic blood flow ($Q_{H}$) and the leaving rate-defining process is fraction unbound in blood ($f_{uB}$) multiplied by the hepatic intrinsic clearance ($CL_{int}$), and thus hepatic clearance ($CL_{H}$) is given by Eq. (1)

(1) $$\frac{1}{CL_{H}}=\frac{1}{Q_{H}}+\frac{1}{f_{uB}\cdot CL_{int}}$$

Solving Eq. (1) gives what has been considered for the past 50 years to be the well-stirred model (WSM). However, Eq. (2) was derived independent of any mechanistic model.


(2) $$CL_{H}=Q_{H}\cdot \frac{f_{uB}\cdot CL_{int}}{Q_{H}+f_{uB}\cdot CL_{int}}$$

Note that regardless of how many exponentials are required to fit the drug concentration-time curve following an iv bolus dose representing multiple distribution sites, passive permeability parameters will never be a rate-defining process. Clearance is never equal to a passive permeability parameter under any conditions and consequently passive permeability meets none of the three rate-defining process criteria. Passive permeability cannot on its own define clearance, it cannot be measured experimentally following an iv bolus dose and the difference in directional passive permeability rate constants or clearances is zero, not a positive value.

When hepatic basolateral transport is clinically relevant, a third rate-defining process must be added, ($PS_{influx}-PS_{efflux}$), where these parameters represent the intrinsic clearance related to passage across the basolateral hepatic membrane, as given in Eq. (3).


(3) $$\frac{1}{CL_{H}}=\frac{1}{Q_{H}}+\frac{1}{f_{uB}\cdot (PS_{influx}-PS_{efflux})}+\frac{1}{f_{uB}\cdot CL_{int}}$$

Solving Eq. (3) when $Q_{H}$ is very much greater than the two other rate-defining processes leads to Eq. (4), as we previously reported (Patcher et al., 2022; Benet and Sodhi, 2023; 2024b; 2024c)

(4) $$CL_{H}=\frac{f_{uB}\cdot (PS_{influx}-PS_{efflux})}{1+\frac{\left(PS_{influs}-PS_{efflux}\right)}{CL_{int}}}$$

Note that the approach to including membrane passage in the hepatic clearance derivation above follows the same approach as for renal clearance ($CL_{R}$), with the only difference being that the membrane passage parameters are included in the $CL_{R}$ equation together with filtration clearance, *i.e.*, the product of fraction unbound in blood and the glomerular filtration rate (GFR).


(5) $$CL_{R}=f_{uB}\cdot GFR+f_{uB}\cdot (PS_{renal\;secretion}-PS_{real\;reabsorption})$$

*In vivo* tubular drug concentrations are not measured for the $CL_{R}$ calculation, nor are liver concentrations measured for the $CL_{H}$ calculation, despite Sugano (2024) extensively discussing the various intercompartmental liver concentrations that are unmeasurable and not relevant for clinical practice. Both $CL_{H}$ and $CL_{R}$ are determined experimentally based only on the amount eliminated divided by *AUC*, the exposure driving that elimination (Benet et al., 2021). The *AUC* value is determined solely from systemic concentration measures. In future publications, we will address the errors being made in estimating active *PS* parameters in both the kidney and liver, specifically the failure to consider the relevance of organ blood flow when it should not be ignored.

## 2. Responding to the Sugano commentary

Recently, Sugano (2024) commented on our publication in EJPS (Benet and Sodhi, 2024a) indicating that he had been a manuscript reviewer. In the Acknowledgements, Sugano wrote that we “kindly suggested publishing his reviewer report as an article”. What we had actually written was: “prior to accepting the [unidentified] reviewer’s approach, however, we believe he/she would have to publish a paper justifying the methodology and proposing how *in vivo* clinical data could be analyzed with such methodology”. We further explained in detail the fallacy of the accepted mechanistic models of hepatic elimination in a subsequent manuscript titled “Pharmacokinetic theory must consider published experimental data” (Benet and Sodhi, 2024b), published online a month after the Sugano commentary became available – unfortunately, not in time for the author to review the full explanation before his commentary was published. Our EJPS publication at issue (Benet and Sodhi, 2024a) does not discuss nor make any assumptions regarding how the ratio of unbound drug concentration in the liver to unbound concentration in the systemic circulation ($Kp_{uu}$) should be measured. However, this is the major topic of the Sugano paper, where almost all of the proposed relationships cannot be measured or verified *in vivo*. We agree that liver physiology is complex and have previously highlighted that the conventional $Kp_{uu}$ approach relies on a single unbound drug concentration in the liver, which oversimplifies the driving force concentrations of various processes such as basolateral efflux, biliary elimination, and metabolism (Sodhi et al., 2020a). The purpose of our EJPS paper at issue was to show that the mechanistic models of hepatic elimination (the well-stirred model (WSM) first proposed by Rowland et al. (1973), the parallel tube model (PTM), first presented in pharmacokinetics by Pang and Rowland (1977), and the infinite number of dispersion models (DMs), first presented in pharmacokinetics by Roberts and Rowland (1986)), all yield $Kp_{uu}$ values, based on systemic concentration measurements, that cannot exceed unity and are all related in some manner to $F_{H}$, the hepatic bioavailability. The WSM, PTM and DMs are all only defined in terms of systemic concentration measurements (Li and Jusko, 2022). We thank Professor Sugano for confirming our findings. He then delves extensively into numerous unmeasurable $Kp_{uu}$ *in vivo* relationships proposed to be relevant but are unapproachable in clinical practice. Thus, we do not believe that the commentary (Sugano, 2024) has met our expectation of justifying his methodology and “proposing how *in vivo* clinical data could be analyzed with such methodology”. As we point out (Benet and Sodhi, 2024b; 2024c), the error in the proposed mechanistic hepatic models is assuming that elimination within the liver is unaffected by hepatic blood flow, although measures of clearance based on systemic concentrations will be affected by hepatic blood flow. No chemical engineering models assume that the reaction process within the reactor is independent of flow rate into the reactor, noting that these models form the basis for the hepatic pharmacokinetic disposition models. Together with our finding (Sodhi et al., 2020b) that no experimental isolated perfused rat liver studies unambiguously are best described by the PT and DM models, our EJPS paper (Benet and Sodhi, 2024a) demonstrates the invalidity of the mechanistic explanations for models of hepatic elimination, as the underlying assumptions used to derive these models result in nonsensical outcomes with respect to the value of $Kp_{uu}$ and its apparent relationship with $F_{H}$.

## 3. The extended clearance model (ECM) should not be utilized to analyze basolateral hepatic transporter effects on hepatic clearance

Sugano (2024) concludes that “when considering mechanistic clearance models, it is easier to start with ECM, and then apply assumptions…In ECM, the drug concentrations and clearances in the liver are explicitly defined. With the development of the science of drug membrane transport, ECM has become increasingly used in modern drug discovery and development.” It is correct that “in the ECM drug concentrations and clearances in the liver are explicitly defined “, but as we have pointed out (Benet and Sodhi. 2024b; Benet and Sodhi, 2024c) they are incorrectly defined. As we reviewed (Benet et al., 2018; Benet and Sodhi, 2024b) the derivation of the ECM based on the WSM approach leads to Eq. (6)

(6) $$CL_{H,ECH}=\frac{Q_{H}\cdot PS_{influx}\cdot f_{uB}\cdot CL_{int}}{Q_{H}\cdot (CL_{int}+PS_{efflux})+PS_{influx}\cdot f_{uB}\cdot CL_{int}}$$

When $Q_{H}$ is much greater than $PS_{influx}\cdot f_{uB}\cdot CL_{int}$, which is seldom tested, then Eq. (6) becomes Eq. (7), the usual ECM equation found in the literature (Di et al., 2021)

(7) $$CL_{H,ECM}=\frac{PS_{influx}\cdot f_{uB}\cdot CL_{int}}{\left(CL_{int}+PS_{efflux}\right)}$$

As noted above, the blood flow relevance is rarely tested, but how is this justified when the iv hepatic blood clearance for fluvastatin is reported to be 1134 ml/min (Tse et al., 1992), atorvastatin approximately 1140 ml/min (Gibson et al., 1997), and repaglinide 877 ml/min (Kudo et al., 2013)? When hepatic blood flow is ignored, the calculated clearance related to transporter effects will be underestimated, particularly for very high clearance drugs, confounding any attempts at *in vitro*-*in vivo* extrapolation and prediction of drug-drug transporter interactions.

As we have detailed in Benet and Sodhi (2024b, 2024c) and above, there are at least five deficiencies with the ECM Eq. (7) as compared to the adapted Kirchhoff’s Laws derivation, Eq. (4).

- In Eq. (7), for hepatic basolateral transport to be rate limiting, $PS_{efflux}$ must be zero or negligible, so that $CL_{int}$ in the numerator and denominator cancel. Then $CL_{H,ECM}=f_{uB}\cdot PS_{influx}$. But why should efflux transport need to be zero or negligible for hepatocellular transport to be rate limiting? From Eq. (4), if the positive difference between influx and efflux is much smaller than hepatic intrinsic clearance, then $CL_{H}=f_{uB}\cdot (PS_{influx}-PS_{efflux})$, yielding a more physiologically appropriate outcome.
- From Eq. (7), it is not possible for $CL_{H,ECM}$ to be rate limited by hepatic intrinsic clearance, unless $PS_{influx}$ equals $PS_{efflux}$ (*i.e.*, the condition for passive membrane transport where transporters are not relevant) and the $PS_{influx}$ value is very large compared to the $CL_{int}$ value. In contrast, Eq. (4) is consistent with $CL_{H}=f_{uB}\cdot CL_{int}$ when $CL_{int}$ is much smaller than the positive difference between influx and efflux, requiring no assumption of the equality of $PS_{influx}$ and $PS_{efflux}$.
- Eq. (7) was derived based on the WSM hypothesis where clearance within the liver is assumed to be independent of hepatic blood flow, initially proposed by Siriani and Pang (1997) and later widely adopted by many others, as we reviewed (Benet et al., 2018).
- Determining $Kp_{uu}$ as the ratio of the WSM-hypothesized average unbound liver concentration to the measured unbound systemic blood concentration results in ECM equation values that are always less than unity and always lower than $F_{H}$ (Benet and Sodhi, 2024a).
- As noted above, when only measuring systemic concentrations to determine hepatic clearance, why wouldn’t hepatic organ clearance follow the same approach as kidney organ clearance, where the two membrane passage parameters are evaluated as a difference?

Contrary to the five limitations of Eq. (7) outlined above, the Eq. (4) relationship can simply identify that when $CL_{int}$ is much greater than ($PS_{influx}-PS_{efflux}$), then hepatic basolateral transport is the rate limiting step; when ($PS_{influx}-PS_{efflux}$) is much greater than $CL_{int}$, hepatic elimination is the rate limiting step. Furthermore, Eq. (4) defines the relationship at intermediate positions when both hepatic basolateral transport and hepatic elimination are relevant. As we previously noted (Benet and Sodhi, 2024c) “All of the clinical clearance relationships for statins and other acids with molecular weights great than 400 (*i.e.*, Extended Clearance Classification System classes 1B and 3B as defined by Varma et al., 2015) can be described by Eq. 4.”

## 4. *In vitro* – *in vivo* extrapolation (IVIVE) predictions of *in vivo* hepatic clearance measurements

The commentary provides a decision tree for hepatic clearance classification, primarily based on liver measurements that cannot be experimentally measured *in vivo*. The commentary (Sugano, 2024) proposes that the decision tree can be used to guide the mechanistic clearance models facilitating the correct choices for IVIVE. For the past decade we have examined the theory and experimental methodologies leading to the general under-prediction of *in vivo* clearance using IVIVE methodology, as thoroughly summarized in Sodhi and Benet (2021). Further, we found no significant improvement in IVIVE predictability based on the mechanistic model of hepatic elimination, no significant difference in the predictability between human hepatocytes and human microsomal enzymes, and no significant improvement by the addition of protein to the *in vitro* clearance measure (Kamayama et al., 2022). Probably the most potential significant finding to improve IVIVE predictability are the recent reports of Yan and coworkers (Yan et al., 2023, 2024a, 2024b) determining dynamic plasma protein binding kinetics using an enzyme reporter assay coupled with high-resolution mass spectrometry, recognizing that the use of the WSM in these predictions does not differ from our mechanism-independent Eq. (2).

## 5. Conclusion

We continue to maintain that current mechanistic models inherently rely on unmeasurable liver unbound drug concentrations *in vivo*, compared to measurable *in vivo* systemic unbound drug concentrations, providing further evidence that the mechanistic models do not provide useful information when only systemic drug concentrations are measured. Although the Sugano (2024) commentary does not disagree with our primary point, it recommends that the ECM be utilized to analyze basolateral hepatic transporter effects on hepatic clearance. Here we detail five deficiencies of the ECM and why the equation incorporating the difference in transporter clearances $\left(PS_{influx}-PS_{efflux}\right)$ should be used. Finally, we do not believe the commentary proposed hepatic clearance decision tree can successfully guide IVIVE prediction.

## Data Availability

No data was used for the research described in the article.

## Abbreviation:

$CL_{H}$: hepatic clearance determined based on systemic blood concentrations
$CL_{int}$: intrinsic hepatic clearance
$CL_{R}$: renal clearance determined based on systemic blood concentrations
DM: dispersion model
ECM: Extended Clearance Model
EJPS: European Journal of Pharmaceutical Sciences
$F_{H}$: hepatic bioavailability
$f_{uB}$: fraction of drug unbound in the blood
GFR: glomerular filtration rate
IVIVE: *in vitro-in vivo* extrapolation
$Kp_{uu}$: *in vivo* ratio at steady-state of unbound liver drug concentration to unbound systemic blood concentration
$PS_{efflux}$: intrinsic basolateral efflux clearance
$PS_{influx}$: intrinsic basolateral influx clearance
$PS_{renal\;reabsorption}$: intrinsic renal reabsorption clearance
$PS_{renal\;scretion}$: intrinsic renal secretion clearance
PTM: parallel tube model
$Q_{H}$: hepatic blood flow
WSM: well-stirred model
